# (*Z*)-*N*-(2,6-Di­methyl­phen­yl)-1-[(2-meth­oxy­phen­yl)amino]­methanimine oxide methanol monosolvate

**DOI:** 10.1107/S2414314624009891

**Published:** 2024-10-21

**Authors:** David O. Juma, Sizwe J. Zamisa, Wisdom Munzeiwa, Bernard Omondi

**Affiliations:** a School of Chemistry and Physics, University of KwaZulu Natal, Private Bag X54001, Westville, Durban, 4000, South Africa; b Chemistry Department, Bindura University of Science Education, Private Bag 1020, Bindura, Zimbabwe; University of Aberdeen, United Kingdom

**Keywords:** Zwitterion, unsymmetrical hy­droxy­formamidine, crystal structure

## Abstract

In the title solvate, C_16_H_18_N_2_O_2_·CH_4_O, the dihedral angles between the formamidine backbone and the pendant 2-meth­oxy­phenyl and 2,6-di­methyl­phenyl groups are 14.84 (11) and 81.61 (12)°, respectively. In the crystal, the components are linked by C—H⋯O, O—H⋯O and C—H⋯ π hydrogen bonds, generating a supra­molecular chain that extends along the crystallographic *a-*axis direction.

## Abstract 2

The crystal structure of a zwitterionic unsymmetrical hy­droxy­formamidine methanol monosolvate is reported.

## Graphical abstract

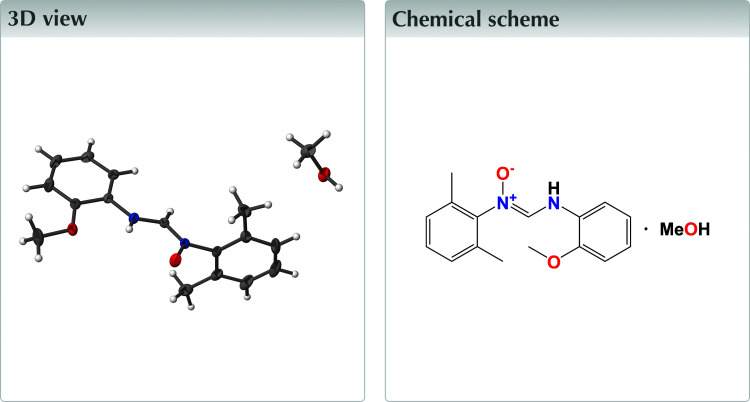

## Structure description

The title compound is categorized in the class of formamidines (Cibian *et al.*, 2011 ▸, Zamisa *et al.*, 2021 ▸). The formamidine backbone features two nitro­gen atoms that provide bidentate coordination sites, making them effective ligands in coordination chemistry (Oshovsky & Pinchuk, 2000 ▸). These metal complexes have demonstrated biological activities such as anti­oxidant (Oladipo *et al.*, 2020 ▸) and anti­bacterial, and significant catalytic activities in the microwave-assisted Suzuki–Miyaura cross-coupling of aryl bromides (Khormi *et al.*, 2019 ▸) and ring-opening polymerization reactions (Akpan *et al.*, 2016 ▸). As part of our studies in this area, we synthesized the title compound, C_16_H_18_N_2_O_2_·CH_4_O, (I), and determined its crystal structure.

**Figure 1:**
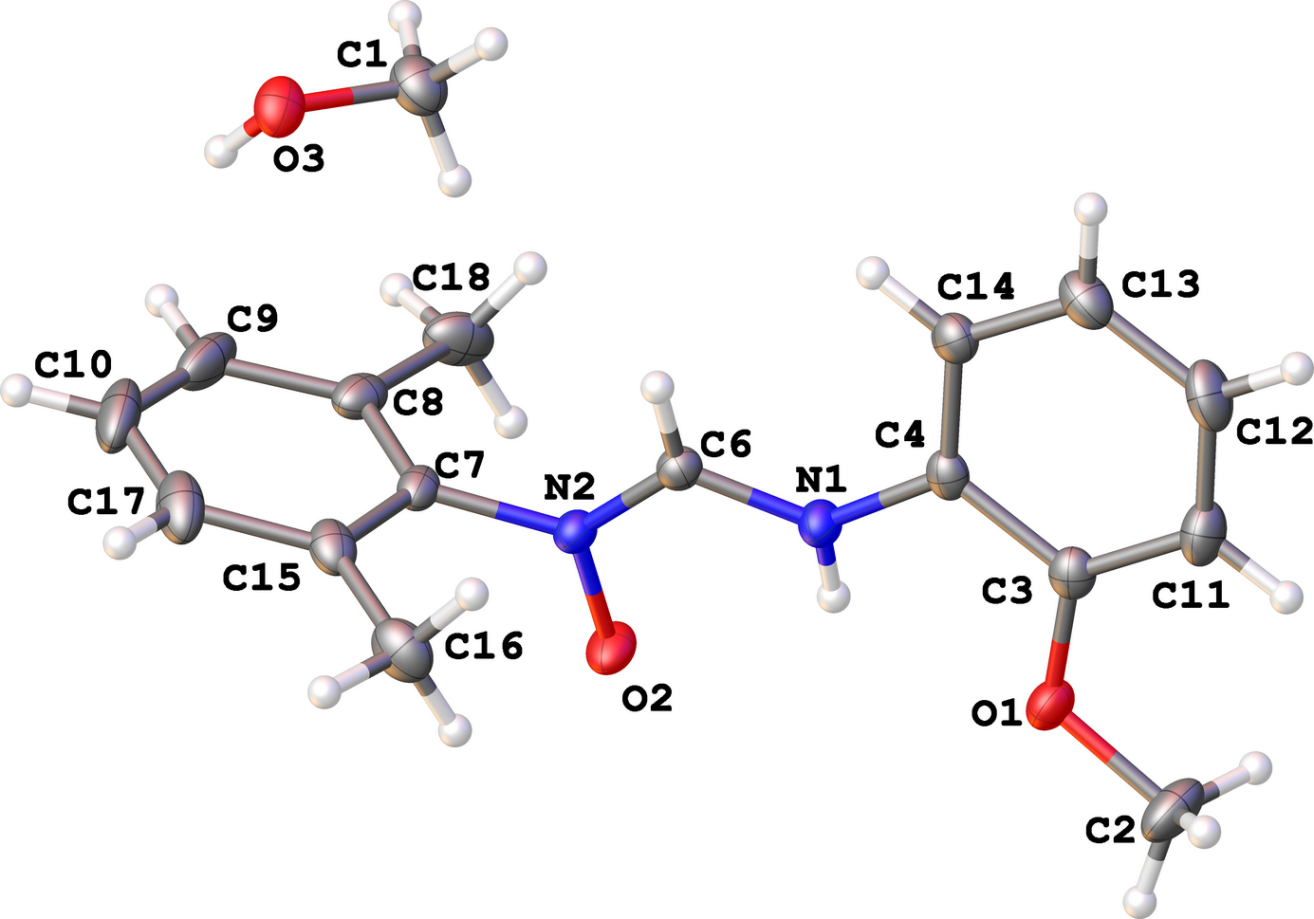
The mol­ecular structure of (I) showing displacement ellipsoid at the 50% probability level.

The asymmetric unit of (I) consists of one substituted formamidine mol­ecule and one methanol solvent mol­ecule as illustrated in Fig. 1 ▸. The mol­ecular structure reveals a non-coplanar arrangement between the formamidine backbone and its pendant phenyl rings with a dihedral angle of 14.84 (11)° between the plane of the C3/C4/C11–C14 2-meth­oxy­phenyl group and the C6/N1/N2/O1 formamidine backbone. In contrast, the dihedral angle between the C7–C10/C15/C17 2,6-di­methyl­phenyl group and the backbone is 81.61 (12)°. The aromatic rings are nearly orthogonal, exhibiting a dihedral angle of 89.25 (5)°.

**Figure 2:**
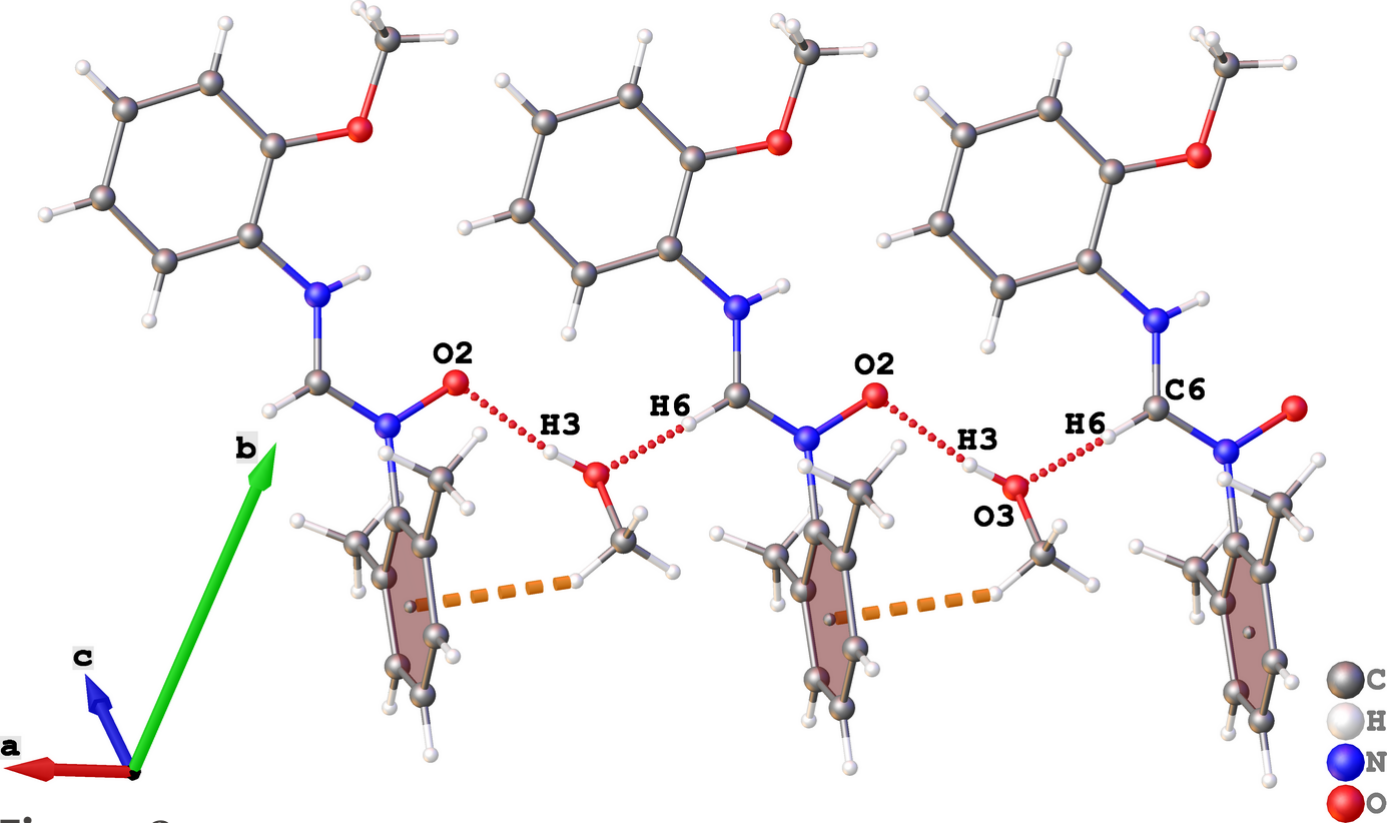
Representation of hydrogen bonds (dotted lines) in the crystal packing of (I).

In the extended structure of (I), C6—H6⋯O3 and O3—H3⋯O2 hydrogen bonds (Table 1 ▸) occur as depicted in Fig. 2 ▸. The former inter­action involves the solvent O atom as acceptor. The latter hydrogen bond involves the methanol OH group as donor and the formamidine O atom as acceptor. Finally, a C—H⋯π inter­action exists between the a methyl H atom of the solvent mol­ecule and the centre of gravity of the di­methyl­phenyl ring (π_DMP_). Together, these generate a one-dimensional supra­molecular structure that extends along the crystallographic *a-*axis direction as shown in Fig. 2 ▸.

## Synthesis and crystallization

The title compound was synthesized following the literature procedure (Munzeiwa *et al.*, 2018 ▸). The crude solid was then recrystallized from methanol solution to produce colourless blocks of (I) suitable for X-ray diffraction.

## Refinement

Crystallographic data and structure refinement details are summarized in Table 2 ▸.

## Supplementary Material

Crystal structure: contains datablock(s) I. DOI: 10.1107/S2414314624009891/hb4489sup1.cif

Structure factors: contains datablock(s) I. DOI: 10.1107/S2414314624009891/hb4489Isup2.hkl

Supporting information file. DOI: 10.1107/S2414314624009891/hb4489Isup3.cml

CCDC reference: 2389686

Additional supporting information:  crystallographic information; 3D view; checkCIF report

## Figures and Tables

**Table 1:** Experimental details

Crystal data	
Chemical formula	C_16_H_18_N_2_O_2_·CH_4_O
*M*_r_	302.36
Crystal system, space group	Monoclinic, *P*2_1_/*n*
Temperature (K)	296
*a*, *b*, *c* (Å)	7.3519 (5), 28.4173 (19), 7.8982 (5)
β (°)	94.481 (2)
*V* (Å^3^)	1645.06 (19)
*Z*	4
Radiation type	Mo *K*α
μ (mm^−1^)	0.08
Crystal size (mm)	0.28 × 0.23 × 0.14

Data collection	
Diffractometer	Bruker APEXII CCD
Absorption correction	–
No. of measured, independent and observed [*I* > 2σ(*I*)] reflections	9891, 3421, 2988
*R*_int_	0.016
(sin θ/λ)_max_ (Å^−1^)	0.635

Refinement	
*R*[*F*^2^ > 2σ(*F*^2^)], *wR*(*F*^2^), *S*	0.038, 0.095, 1.04
No. of reflections	3421
No. of parameters	204
H-atom treatment	H-atom parameters constrained
Δρ_max_, Δρ_min_ (e Å^−3^)	0.24, −0.19

Computer programs: *APEX2* and *SAINT* (Bruker, 2016 ▸), *SHELXL2018/3* (Sheldrick, 2015 ▸) and *OLEX2* (Dolomanov *et al.*, 2009 ▸).

**Table 2:** Hydrogen-bond geometry (Å, °) π is the centroid of the C7–C10/C15/C17 ring.

*D*—H⋯*A*	*D*—H	H⋯*A*	*D*⋯*A*	*D*—H⋯*A*
C6—H6⋯O3^i^	0.95	2.20	3.1134 (14)	160
O3—H3⋯O2^ii^	0.84	1.85	2.6887 (12)	173
C1—H1*C*⋯π^i^	0.98	2.66	3.5182 (16)	148

Symmetry codes: (i) 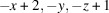; (ii) 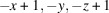.

## full crystallographic data

### (Z)-N-(2,6-Dimethylphenyl)-1-[(2-methoxyphenyl)amino]methanimine oxide methanol monosolvate Crystal data

**Table:**

C_16_H_18_N_2_O_2_·CH_4_O	*F*(000) = 648
*M**_r_* = 302.36	*D*_x_ = 1.221 Mg m^−^^3^
Monoclinic, *P*2_1_/*n*	Mo *K*α radiation, λ = 0.71073 Å
*a* = 7.3519 (5) Å	Cell parameters from 5449 reflections
*b* = 28.4173 (19) Å	θ = 2.9–26.8°
*c* = 7.8982 (5) Å	µ = 0.08 mm^−^^1^
β = 94.481 (2)°	*T* = 296 K
*V* = 1645.06 (19) Å^3^	Block, colourless
*Z* = 4	0.28 × 0.23 × 0.14 mm

### (Z)-N-(2,6-Dimethylphenyl)-1-[(2-methoxyphenyl)amino]methanimine oxide methanol monosolvate Data collection

**Table:**

Bruker APEXII CCD diffractometer	*R*_int_ = 0.016
Graphite monochromator	θ_max_ = 26.8°, θ_min_ = 2.7°
φ and ω scans	*h* = −5→9
9891 measured reflections	*k* = −35→29
3421 independent reflections	*l* = −10→7
2988 reflections with *I* > 2σ(*I*)	

### (Z)-N-(2,6-Dimethylphenyl)-1-[(2-methoxyphenyl)amino]methanimine oxide methanol monosolvate Refinement

**Table:**

Refinement on *F*^2^	Primary atom site location: dual
Least-squares matrix: full	Hydrogen site location: inferred from neighbouring sites
*R*[*F*^2^ > 2σ(*F*^2^)] = 0.038	H-atom parameters constrained
*wR*(*F*^2^) = 0.095	*w* = 1/[σ^2^(*F*_o_^2^) + (0.0379*P*)^2^ + 0.7025*P*] where *P* = (*F*_o_^2^ + 2*F*_c_^2^)/3
*S* = 1.04	(Δ/σ)_max_ < 0.001
3421 reflections	Δρ_max_ = 0.24 e Å^−^^3^
204 parameters	Δρ_min_ = −0.19 e Å^−^^3^
0 restraints	

### (Z)-N-(2,6-Dimethylphenyl)-1-[(2-methoxyphenyl)amino]methanimine oxide methanol monosolvate Special details

**Table:**

Geometry. All esds (except the esd in the dihedral angle between two l.s. planes) are estimated using the full covariance matrix. The cell esds are taken into account individually in the estimation of esds in distances, angles and torsion angles; correlations between esds in cell parameters are only used when they are defined by crystal symmetry. An approximate (isotropic) treatment of cell esds is used for estimating esds involving l.s. planes.

### (Z)-N-(2,6-Dimethylphenyl)-1-[(2-methoxyphenyl)amino]methanimine oxide methanol monosolvate Fractional atomic coordinates and isotropic or equivalent isotropic displacement parameters (Å^2^)

**Table:**

	*x*	*y*	*z*	*U*_iso_*/*U*_eq_	
O1	0.95025 (12)	0.23202 (3)	0.86095 (11)	0.0234 (2)	
O2	0.68659 (11)	0.13040 (3)	0.54807 (11)	0.0253 (2)	
N1	0.99859 (13)	0.15522 (4)	0.69356 (13)	0.0196 (2)	
H1	0.891940	0.165508	0.721129	0.023*	
N2	0.83888 (13)	0.11068 (3)	0.49709 (12)	0.0172 (2)	
C2	0.9131 (2)	0.27445 (5)	0.9498 (2)	0.0360 (4)	
H2A	0.929940	0.268838	1.072488	0.054*	
H2B	0.787040	0.284311	0.919371	0.054*	
H2C	0.996864	0.299228	0.918477	0.054*	
C3	1.12632 (17)	0.21578 (4)	0.87530 (15)	0.0204 (3)	
C4	1.15367 (16)	0.17426 (4)	0.78411 (14)	0.0183 (2)	
C6	0.99662 (16)	0.12294 (4)	0.56934 (15)	0.0177 (2)	
H6	1.106504	0.109551	0.535504	0.021*	
C7	0.82105 (15)	0.07864 (4)	0.35527 (15)	0.0190 (3)	
C8	0.77144 (16)	0.03222 (5)	0.38559 (18)	0.0252 (3)	
C9	0.74859 (19)	0.00278 (5)	0.2439 (2)	0.0362 (4)	
H9	0.714657	−0.029146	0.258361	0.043*	
C10	0.7747 (2)	0.01942 (6)	0.0827 (2)	0.0407 (4)	
H10	0.759648	−0.001348	−0.011605	0.049*	
C11	1.27136 (19)	0.23666 (5)	0.96940 (17)	0.0273 (3)	
H11	1.253247	0.264546	1.032260	0.033*	
C12	1.44422 (19)	0.21639 (5)	0.97109 (17)	0.0299 (3)	
H12	1.543994	0.230569	1.035715	0.036*	
C13	1.47188 (17)	0.17590 (5)	0.87973 (16)	0.0265 (3)	
H13	1.590666	0.162711	0.880300	0.032*	
C14	1.32620 (17)	0.15440 (5)	0.78691 (15)	0.0214 (3)	
H14	1.344791	0.126243	0.725748	0.026*	
C15	0.84516 (16)	0.09678 (5)	0.19363 (16)	0.0241 (3)	
C16	0.89151 (19)	0.14775 (5)	0.16957 (17)	0.0309 (3)	
H16A	0.884152	0.155090	0.047995	0.046*	
H16B	1.015574	0.153864	0.219298	0.046*	
H16C	0.805035	0.167507	0.225731	0.046*	
C17	0.82203 (19)	0.06565 (6)	0.05721 (18)	0.0347 (3)	
H17	0.839093	0.076404	−0.054328	0.042*	
C18	0.7448 (2)	0.01537 (5)	0.5626 (2)	0.0330 (3)	
H18A	0.695056	−0.016641	0.557656	0.050*	
H18B	0.659657	0.036362	0.615160	0.050*	
H18C	0.862325	0.015410	0.630341	0.050*	
O3	0.63925 (12)	−0.10123 (3)	0.59154 (11)	0.0256 (2)	
H3	0.533296	−0.108674	0.553427	0.038*	
C1	0.64434 (18)	−0.09642 (5)	0.76969 (17)	0.0300 (3)	
H1A	0.765090	−0.085163	0.813127	0.045*	
H1B	0.620195	−0.126986	0.820810	0.045*	
H1C	0.551275	−0.073758	0.798853	0.045*	

### (Z)-N-(2,6-Dimethylphenyl)-1-[(2-methoxyphenyl)amino]methanimine oxide methanol monosolvate Atomic displacement parameters (Å^2^)

**Table:**

	*U*^11^	*U*^22^	*U*^33^	*U*^12^	*U*^13^	*U*^23^
O1	0.0260 (5)	0.0184 (5)	0.0257 (5)	0.0002 (4)	0.0019 (4)	−0.0049 (4)
O2	0.0162 (4)	0.0293 (5)	0.0304 (5)	0.0031 (4)	0.0026 (4)	−0.0106 (4)
N1	0.0170 (5)	0.0210 (5)	0.0205 (5)	−0.0002 (4)	0.0003 (4)	−0.0037 (4)
N2	0.0166 (5)	0.0168 (5)	0.0185 (5)	0.0007 (4)	0.0021 (4)	−0.0021 (4)
C2	0.0344 (8)	0.0243 (7)	0.0503 (9)	−0.0006 (6)	0.0086 (7)	−0.0147 (7)
C3	0.0235 (6)	0.0200 (6)	0.0180 (6)	−0.0033 (5)	0.0027 (5)	0.0017 (5)
C4	0.0216 (6)	0.0189 (6)	0.0144 (5)	−0.0040 (5)	0.0008 (4)	0.0021 (4)
C6	0.0183 (5)	0.0165 (6)	0.0185 (6)	−0.0002 (5)	0.0019 (4)	0.0003 (4)
C7	0.0127 (5)	0.0213 (6)	0.0229 (6)	0.0011 (4)	−0.0001 (4)	−0.0066 (5)
C8	0.0168 (6)	0.0213 (6)	0.0369 (7)	0.0019 (5)	−0.0015 (5)	−0.0044 (5)
C9	0.0250 (7)	0.0239 (7)	0.0588 (10)	0.0003 (6)	−0.0032 (6)	−0.0180 (7)
C10	0.0286 (7)	0.0509 (10)	0.0420 (9)	0.0018 (7)	−0.0005 (6)	−0.0304 (8)
C11	0.0319 (7)	0.0255 (7)	0.0246 (7)	−0.0082 (6)	0.0031 (5)	−0.0062 (5)
C12	0.0258 (7)	0.0395 (8)	0.0238 (7)	−0.0119 (6)	−0.0026 (5)	−0.0037 (6)
C13	0.0208 (6)	0.0369 (8)	0.0215 (6)	−0.0015 (5)	−0.0001 (5)	0.0021 (5)
C14	0.0231 (6)	0.0235 (6)	0.0173 (6)	−0.0001 (5)	0.0007 (5)	0.0010 (5)
C15	0.0148 (6)	0.0353 (7)	0.0220 (6)	−0.0001 (5)	0.0007 (5)	−0.0051 (5)
C16	0.0290 (7)	0.0396 (8)	0.0241 (7)	−0.0030 (6)	0.0024 (5)	0.0067 (6)
C17	0.0240 (7)	0.0541 (10)	0.0262 (7)	−0.0006 (6)	0.0030 (5)	−0.0156 (7)
C18	0.0295 (7)	0.0216 (7)	0.0472 (9)	−0.0033 (6)	−0.0022 (6)	0.0078 (6)
O3	0.0190 (4)	0.0304 (5)	0.0278 (5)	−0.0047 (4)	0.0041 (4)	−0.0061 (4)
C1	0.0239 (6)	0.0385 (8)	0.0273 (7)	−0.0006 (6)	0.0001 (5)	0.0000 (6)

### (Z)-N-(2,6-Dimethylphenyl)-1-[(2-methoxyphenyl)amino]methanimine oxide methanol monosolvate Geometric parameters (Å, º)

**Table:**

O1—C2	1.4323 (15)	C10—C17	1.378 (2)
O1—C3	1.3706 (15)	C11—H11	0.9500
O2—N2	1.3417 (13)	C11—C12	1.394 (2)
N1—H1	0.8800	C12—H12	0.9500
N1—C4	1.4061 (15)	C12—C13	1.382 (2)
N1—C6	1.3423 (15)	C13—H13	0.9500
N2—C6	1.2995 (15)	C13—C14	1.3911 (18)
N2—C7	1.4415 (15)	C14—H14	0.9500
C2—H2A	0.9800	C15—C16	1.503 (2)
C2—H2B	0.9800	C15—C17	1.3943 (19)
C2—H2C	0.9800	C16—H16A	0.9800
C3—C4	1.4049 (17)	C16—H16B	0.9800
C3—C11	1.3847 (18)	C16—H16C	0.9800
C4—C14	1.3870 (17)	C17—H17	0.9500
C6—H6	0.9500	C18—H18A	0.9800
C7—C8	1.3941 (18)	C18—H18B	0.9800
C7—C15	1.4008 (17)	C18—H18C	0.9800
C8—C9	1.397 (2)	O3—H3	0.8400
C8—C18	1.505 (2)	O3—C1	1.4113 (16)
C9—H9	0.9500	C1—H1A	0.9800
C9—C10	1.386 (2)	C1—H1B	0.9800
C10—H10	0.9500	C1—H1C	0.9800
			
C3—O1—C2	117.25 (10)	C12—C11—H11	120.3
C4—N1—H1	116.7	C11—C12—H12	119.7
C6—N1—H1	116.7	C13—C12—C11	120.68 (12)
C6—N1—C4	126.64 (10)	C13—C12—H12	119.7
O2—N2—C7	118.01 (9)	C12—C13—H13	119.9
C6—N2—O2	119.55 (10)	C12—C13—C14	120.15 (12)
C6—N2—C7	122.31 (10)	C14—C13—H13	119.9
O1—C2—H2A	109.5	C4—C14—C13	119.68 (12)
O1—C2—H2B	109.5	C4—C14—H14	120.2
O1—C2—H2C	109.5	C13—C14—H14	120.2
H2A—C2—H2B	109.5	C7—C15—C16	121.06 (11)
H2A—C2—H2C	109.5	C17—C15—C7	117.02 (13)
H2B—C2—H2C	109.5	C17—C15—C16	121.92 (13)
O1—C3—C4	114.35 (10)	C15—C16—H16A	109.5
O1—C3—C11	125.65 (11)	C15—C16—H16B	109.5
C11—C3—C4	120.00 (12)	C15—C16—H16C	109.5
C3—C4—N1	116.18 (11)	H16A—C16—H16B	109.5
C14—C4—N1	123.75 (11)	H16A—C16—H16C	109.5
C14—C4—C3	120.07 (11)	H16B—C16—H16C	109.5
N1—C6—H6	121.2	C10—C17—C15	120.59 (14)
N2—C6—N1	117.55 (11)	C10—C17—H17	119.7
N2—C6—H6	121.2	C15—C17—H17	119.7
C8—C7—N2	118.24 (11)	C8—C18—H18A	109.5
C8—C7—C15	123.93 (12)	C8—C18—H18B	109.5
C15—C7—N2	117.74 (11)	C8—C18—H18C	109.5
C7—C8—C9	116.49 (13)	H18A—C18—H18B	109.5
C7—C8—C18	121.02 (12)	H18A—C18—H18C	109.5
C9—C8—C18	122.49 (13)	H18B—C18—H18C	109.5
C8—C9—H9	119.5	C1—O3—H3	109.5
C10—C9—C8	121.00 (14)	O3—C1—H1A	109.5
C10—C9—H9	119.5	O3—C1—H1B	109.5
C9—C10—H10	119.5	O3—C1—H1C	109.5
C17—C10—C9	120.96 (13)	H1A—C1—H1B	109.5
C17—C10—H10	119.5	H1A—C1—H1C	109.5
C3—C11—H11	120.3	H1B—C1—H1C	109.5
C3—C11—C12	119.41 (12)		
			
O1—C3—C4—N1	−0.57 (15)	C6—N1—C4—C14	−16.25 (19)
O1—C3—C4—C14	179.46 (10)	C6—N2—C7—C8	102.55 (13)
O1—C3—C11—C12	−179.40 (12)	C6—N2—C7—C15	−80.82 (14)
O2—N2—C6—N1	0.11 (16)	C7—N2—C6—N1	175.94 (10)
O2—N2—C7—C8	−81.55 (14)	C7—C8—C9—C10	0.08 (19)
O2—N2—C7—C15	95.08 (13)	C7—C15—C17—C10	1.02 (19)
N1—C4—C14—C13	179.74 (11)	C8—C7—C15—C16	177.54 (12)
N2—C7—C8—C9	177.55 (11)	C8—C7—C15—C17	−1.69 (18)
N2—C7—C8—C18	−2.53 (17)	C8—C9—C10—C17	−0.7 (2)
N2—C7—C15—C16	1.12 (17)	C9—C10—C17—C15	0.1 (2)
N2—C7—C15—C17	−178.12 (11)	C11—C3—C4—N1	179.35 (11)
C2—O1—C3—C4	−179.56 (11)	C11—C3—C4—C14	−0.62 (18)
C2—O1—C3—C11	0.52 (18)	C11—C12—C13—C14	−1.1 (2)
C3—C4—C14—C13	−0.30 (18)	C12—C13—C14—C4	1.14 (19)
C3—C11—C12—C13	0.2 (2)	C15—C7—C8—C9	1.15 (18)
C4—N1—C6—N2	−178.00 (11)	C15—C7—C8—C18	−178.94 (12)
C4—C3—C11—C12	0.69 (19)	C16—C15—C17—C10	−178.21 (13)
C6—N1—C4—C3	163.79 (11)	C18—C8—C9—C10	−179.84 (13)

### (Z)-N-(2,6-Dimethylphenyl)-1-[(2-methoxyphenyl)amino]methanimine oxide methanol monosolvate Hydrogen-bond geometry (Å, º)

π is the centroid of the C7–C10/C15/C17 ring.

**Table:**

*D*—H···*A*	*D*—H	H···*A*	*D*···*A*	*D*—H···*A*
C6—H6···O3^i^	0.95	2.20	3.1134 (14)	160
O3—H3···O2^ii^	0.84	1.85	2.6887 (12)	173
C1—H1*C*···π^i^	0.98	2.66	3.5182 (16)	148

Symmetry codes: (i) −*x*+2, −*y*, −*z*+1; (ii) −*x*+1, −*y*, −*z*+1.
